# Errata in East Coast tick study: retort to Tufts & Diuk-Wasser

**DOI:** 10.1186/s13071-021-05096-4

**Published:** 2021-11-25

**Authors:** John D. Scott

**Affiliations:** Upper Grand Tick Centre, Fergus, ON N1M 2L7 Canada

**Keywords:** Ticks, *Haemaphysalis punctata*, Climate change

## Abstract

The authors overlook the first report of *Haemaphysalis punctata* in the Western Hemisphere documented by a pioneer acarologist in 1910. The authors assume that climate change alters movement of ticks, but provide no data. The authors’ assumptions are only opinions, and must be corrected and challenged.

Letter to the Editor,

Tufts & Diuk-Wasser purport that their discovery of the red sheep tick, *Haemaphysalis punctata* (Acari: Ixodidae), on Block Island, Rhode Island, USA, is the first report of this tick species in the Western Hemisphere [1]. However, Hadwen reported *H. punctata* at Winnipeg, Manitoba 111 years earlier [2]. Moreover, *H. punctata* was reported by C.L. Koch, at Para, Brazil in 1847. Therefore, Tufts and Diuk-Wasser have overlooked previously published scientific research on the presence of *H. punctata* in the Western Hemisphere.

Tufts & Diuk-Wasser allege that range expansion of ticks is due to climate change. In fact, the ambient temperatures on Block Island are modulated by the Gulf Stream, and there are no data (i.e. historical daily mean temperatures over the past 100 years) to substantiate climate change on this coastal island. In reality, multiple abiotic and biotic factors contribute to the distribution of *H. punctata*, including dog travel, suitable hosts, photoperiod, songbird migration, livestock imports, seasonal weather variation and dislocation of parasitized songbirds during trans-Atlantic storms. Based on a recent Scandinavian tick-host study [3], researchers on bird ticks reported *H. punctata* parasitizing songbirds, and these avian hosts have the potential to widely disperse *H. punctata*, especially during bidirectional migration. In North America, passerine migrants disperse ticks, particularly during northward spring migration [4]. Pertinent to native ticks, the blacklegged tick, *Ixodes scapularis*, which is indigenous east of the Rocky Mountains, is eco-adaptive. For example, at Kenora, Ontario, this tick species survives temperatures ranging from − 44 °C to + 36 °C. This is a temperature differential of 80 °C. Any research to link ticks to climate change has been inconclusive and unsubstantiated.

Not only did the authors miss the initial discovery of *H. punctata* in North America, they unfortunately failed to justify any finite effect of climate change on ticks.
